# Structural and Dynamic Requirements for Optimal Activity of the Essential Bacterial Enzyme Dihydrodipicolinate Synthase

**DOI:** 10.1371/journal.pcbi.1002537

**Published:** 2012-06-07

**Authors:** C. F. Reboul, B. T. Porebski, M. D. W. Griffin, R. C. J. Dobson, M. A. Perugini, J. A. Gerrard, A. M. Buckle

**Affiliations:** 1 Department of Biochemistry and Molecular Biology, Monash University, Clayton, Victoria, Australia; 2 ARC Centre of Excellence in Structural and Functional Microbial Genomics, Monash University, Clayton, Victoria, Australia; 3 Department of Biochemistry and Molecular Biology, Bio21 Molecular Science and Biotechnology Institute, The University of Melbourne, Parkville, Victoria, Australia; 4 Department of Biochemistry, La Trobe Institute for Molecular Science, La Trobe University, Melbourne, Victoria, Australia; 5 Biomolecular Interaction Centre, and School of Biological Sciences, University of Canterbury, Christchurch, New Zealand; University of Houston, United States of America

## Abstract

Dihydrodipicolinate synthase (DHDPS) is an essential enzyme involved in the lysine biosynthesis pathway. DHDPS from E. coli is a homotetramer consisting of a ‘dimer of dimers’, with the catalytic residues found at the tight-dimer interface. Crystallographic and biophysical evidence suggest that the dimers associate to stabilise the active site configuration, and mutation of a central dimer-dimer interface residue destabilises the tetramer, thus increasing the flexibility and reducing catalytic efficiency and substrate specificity. This has led to the hypothesis that the tetramer evolved to optimise the dynamics within the tight-dimer. In order to gain insights into DHDPS flexibility and its relationship to quaternary structure and function, we performed comparative Molecular Dynamics simulation studies of native tetrameric and dimeric forms of DHDPS from E. coli and also the native dimeric form from methicillin-resistant Staphylococcus aureus (MRSA). These reveal a striking contrast between the dynamics of tetrameric and dimeric forms. Whereas the E. coli DHDPS tetramer is relatively rigid, both the E. coli and MRSA DHDPS dimers display high flexibility, resulting in monomer reorientation within the dimer and increased flexibility at the tight-dimer interface. The mutant E. coli DHDPS dimer exhibits disorder within its active site with deformation of critical catalytic residues and removal of key hydrogen bonds that render it inactive, whereas the similarly flexible MRSA DHDPS dimer maintains its catalytic geometry and is thus fully functional. Our data support the hypothesis that in both bacterial species optimal activity is achieved by fine tuning protein dynamics in different ways: E. coli DHDPS buttresses together two dimers, whereas MRSA dampens the motion using an extended tight-dimer interface.

## Author Summary

Enzyme function requires the specific placement of residues in the active site so that the correct chemistry is available for efficient catalysis. However, the inherent flexibility of proteins can present challenges in fulfilling these stringent requirements. We have investigated the role of flexibility in the enzyme Dihydrodipicolinate synthase (DHDPS), which in E. coli is a homotetramer consisting of a ‘dimer of dimers’, with the catalytic residues found at the tight-dimer interface. It is hypothesized that the tetramer arrangement has evolved to restrict the flexibility at the active site by buttressing together a pair of dimers. In contrast, DHDPS from methicillin resistant Staphylococcus aureus (MRSA) occurs naturally as a dimer yet retains full activity. Using molecular dynamics simulations we have investigated the flexibility of dimeric and tetrameric forms of the *E. coli* and MR*SA* enzymes, and reveal that optimal activity is achieved by minimizing the inherent dimer flexibility using two different strategies – by either buttressing two dimers together in the case of the E. coli tetrameric enzyme or strengthening and extending the dimer interface in the dimeric MRSA.

## Introduction

**Figure 1:**
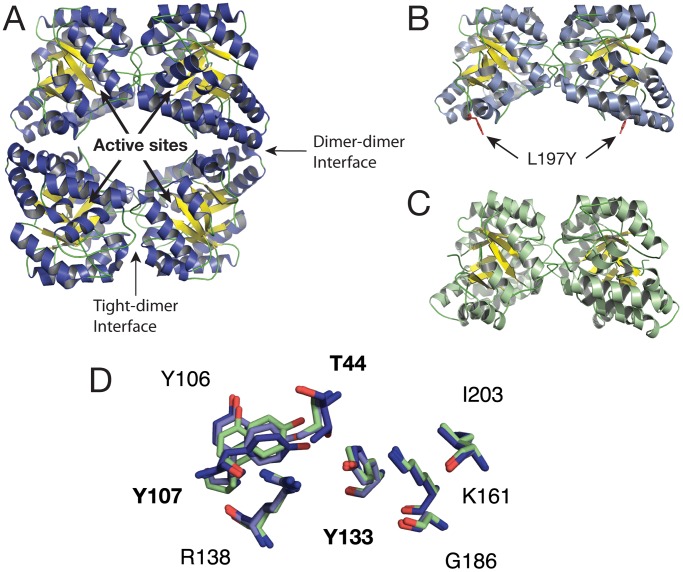
Cartoon representations of DHDPS crystallographic structures. (A) Wild-type *E. coli*; (B) *E. coli* L197Y mutant dimer; (C) wild-type MR*SA* dimer. The arrows indicate locations of the active sites (1 per monomer) and tight-dimer interfaces; (D) Active site alignments of tetramer and dimers. Wild-type *E. coli* tetramer (dark blue), *E. coli* L197 mutant dimer (light blue), MR*SA* wild-type dimer (green).

Dihydrodipicolinate synthase (DHDPS) is an essential enzyme involved in the lysine biosynthesis pathway [1]. It is expressed in plants and microorganisms, but not in animals, which makes it a potential target for herbicides and antibiotics [2]. DHDPS from E. coli is a homotetramer consisting of a ‘dimer of dimers’ (Figure 1A). The catalytic residues T44, Y107 and Y133 are found at the tight-dimer interface (Figure 1D), with each tight-dimer containing two complete active sites within the barrel of the monomeric (β/α)_8_-fold and an allosteric site within a deep cleft between the subunits that binds two (S)-lysine molecules to mediate feedback inhibition [3]. A tyrosine residue (Y107) from one subunit of the tight-dimer protrudes into the active site of the adjacent subunit and forms part of a catalytic triad that is essential for activity [4], [5]. Although this suggests that the tight-dimer contains the minimum requirements for catalysis, mutation of a central residue in the dimer–dimer interface (L197) produced dimeric variants having severely reduced catalytic function (Figure 1B) [6], [7]. Crystallographic, biophysical and Small Angle X-ray Scattering (SAXS) evidence suggest that the dimers associate to stabilise the active site configuration, and removal of this central interface residue destabilises the dimer, thus increasing the flexibility and reducing both catalytic efficiency and substrate specificity. This has led to the hypothesis that the tetramer has evolved to optimise the dynamics within the tight-dimer unit [6].

Interestingly, DHDPS from methicillin resistant Staphylococcus aureus (MRSA) occurs naturally as a dimer [8] and contains a significantly more extensive tight-dimer interface compared to DHDPS from other species (Figure 1C). It has been suggested [8] that this serves to restrict flexibility at the interface, and represents an alternate evolutionary solution to optimising dynamics across this interface and thus enzyme activity.

Although the crystal structures for DHDPS from over 20 species have been determined to date, and together with biophysical and biochemical data have provided insight into the role of quaternary structure in regulating DHDPS activity, a detailed molecular understanding of the conformational properties of dimeric and tetrameric forms of DHDPS has not yet emerged. While X-ray crystallography is a powerful technique for understanding protein structure at atomic resolution, the final model represents a space and time average of all molecules in the crystal lattice. Therefore information about the flexibility of the molecule is limited and can only be gained from structural comparisons of the molecule in different crystal lattices or the atomic temperature (B) factors; although these values must be interpreted with caution. Insights into flexibility and motion can be obtained using the X-ray crystal structure combined with molecular dynamics (MD) simulations. This offers the ability to study the time-dependent behaviour of a molecular system, extending the information gained from crystallographic and other data. In this study, we take a unique opportunity to probe the role of quaternary structure in enzyme catalysis using three well-characterised forms of DHDPS. We perform comparative MD simulation studies of native tetrameric, mutant dimeric forms of DHDPS from E. coli and the native dimeric structure from MRSA, with the aim of understanding the importance of quaternary structure to the dynamics and function of this essential enzyme.

## Results/Discussion

### Disruption of the E. coli DHDPS dimer-dimer interface affects overall flexibility

To probe the dynamic features of both tetrameric and dimeric forms of E. coli DHDPS, we performed comparative MD simulations of the wild-type E. coli tetramer (referred to as tet-1 and tet-2; simulated for 0.48 µs each) and E. coli dimer (dim-A = L197Y mutant dimer; dim-B = dimer taken from the wild-type tetramer; 0.5 µs each) in the absence of substrate.

**Figure 2:**
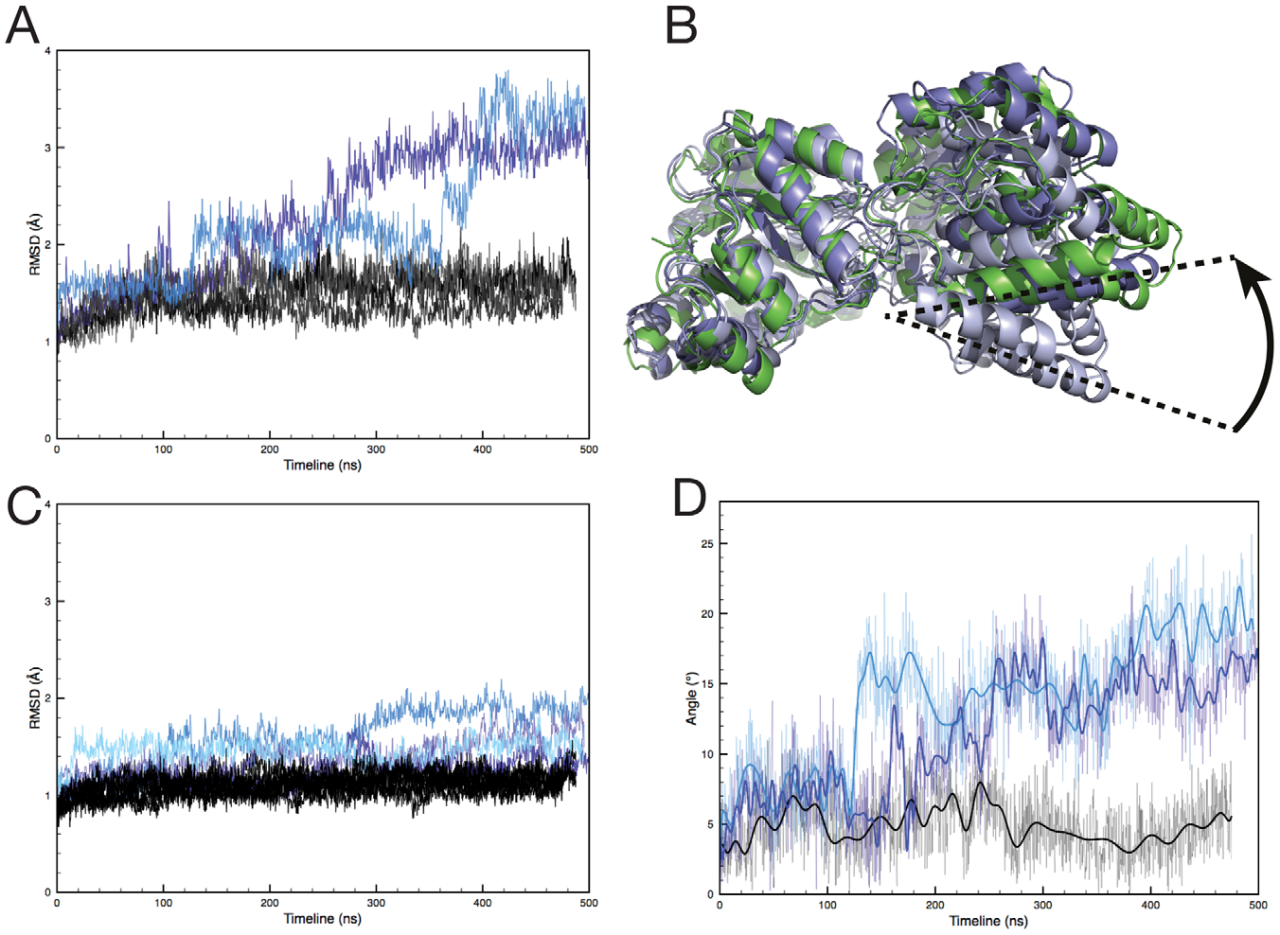
Overall simulations results for *E. coli* DHDPS tetramer and dimer. (A) Cα RMSDs over the course of the simulations, for dimers from tet-1 & tet-2 (shades of grey), dim-A (blue), dim-B (light blue); (B) Cartoon representation of monomer-monomer reorientation during simulation of dimers. The relative rotation of monomers is represented by dotted lines and an arrow. Cartoons are shown for extreme conformations taken from dim-B (light-blue at 70 ns, blue at 433 ns), and mrsa-1 (green at 430 ns). Cα RMSD between extreme conformations are: 4.0 Å for the *E.coli* and 3.8 Å for the MR*SA* dimers. (C) Cα RMSD values for monomers from tet-1 & tet-2 (shades of grey), dim-A & dim-B (shades of blue); (D) Angles of rotation corresponding to monomer rearrangement. Only tet1-A (black), dim-A (blue) and dim-B (light blue) are represented for clarity, the thick lines represent the spline fit of the values.

Both tetramer simulations consistently exhibited steady dynamics and reached an RMSD plateau from 80 ns until the end of the simulations with an RMSD = 1.5 Å, only slightly deviating from the crystal structure conformation (Figure 2A, grey lines; Video S1). In comparison the L197Y mutant dimer simulation (dim-A) showed a strikingly different behaviour (Figure 2A, blue; Video S2). While the Cα−RMSD curve remained close to the tetramer simulations for the first 150 ns, it increased to reach a RMSD plateau at ∼3.1 Å for the last 200 ns of simulation. Closer examination revealed that the increase in RMSD is largely a result of the 15 degrees relative re-orientation of monomers within the dimer (Figure 2B). RMSDs of Cα atoms within individual monomers in dim-A remained low throughout the simulations (mean RMSD ∼1.5 Å, Figure 2C), comparable to the steady RMSDs observed in all monomers simulations of tet-1 and tet-2 (mean RMSD = 1.1 Å). This indicates that the monomers experience relatively little structural deviation from their crystal conformation individually in dim-A, but undergo significant rigid-body motion, relative to each other, within the dimer. The angle of rotation of the monomers for the dim-A simulation is represented in Figure 2D (blue).

Consistent with the dim-A simulation, dim-B Cα-RMSDs remained close to those from the tetramer for the first 130 ns, then increased to ∼2.1 Å for 220 ns to reach a final plateau for the last 100 ns of the simulation at 3.3 Å (Figure 2A, light blue), only slightly above the value reached by dim-A and well above the RMSDs of the tetramer simulations. Again, the increase in the RMSDs can be explained by monomer-monomer rotation (Figure 2D, light blue), with the Cα-RMSDs within each monomer remaining low throughout the simulation (1 to 1.8 Å; Figure 2C).

Taken together, these simulations indicate that the dimer produced by disrupting the dimer-dimer interface of the native E. coli DHDPS tetramer, either as a result of the L197Y mutation or by artificially splitting the wild-type tetramer in half, loses the stabilising contribution of its adjacent dimer. Similar results have recently been obtained from MD simulations for DHDPS from the plant species, Vitis Vinifera [9], which forms a ‘back-to-back’ dimer of dimers compared to the head-to-head arrangement of E. coli DHDPS (Figure 1A). Despite the different quaternary architecture, the loss of dimer-dimer packing in the plant or bacterial tetramers also results in monomers moving more freely within the dimer. Further, SAXS studies of the E. coli mutant dimer [6] used in this work have suggested rigid-body motion of the monomers within the dimer and are thus consistent with our observations. As this motion revolves around the tight-dimer interface that also comprises some of the important active site residues, we next focused on comparing the nature and extent of active site flexibility in E. coli DHDPS tetramers and dimers.

### Active site flexibility and deformation in the E. coli dimer

**Figure 3:**
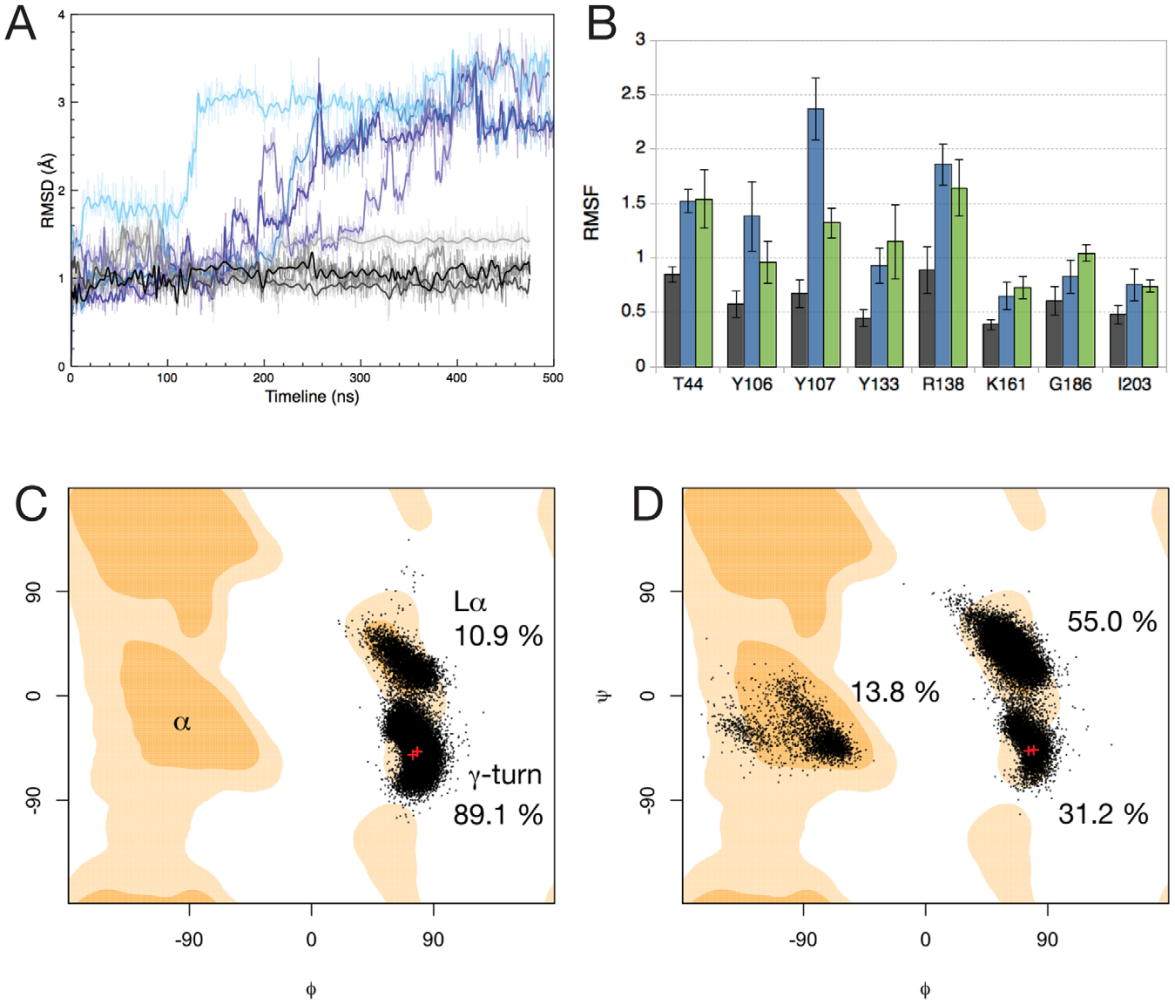
Flexibility and stereochemistry of active sites in *E. coli* DHDPS tetramer and dimer simulations. (A) RMSDs of active site residues for *E. coli* tetramers and dimers: tet-1 & tet-2 (grey shades, 8 curves overlayed for 2×4 active sites), dim-A (light blue, 2 curves), dim-B (dark blue, 2 curves). (B) Individual RMSFs of active site residues, averaged over all simulations, with error bars: tet-1 and tet-2 (grey, 2×4 active sites), dim-A and dim-B (blue, 2×2 active sites), mrsa-1 and mrsa-2 (green, 2×2 active sites; *E*. coli numbering). (C) and (D) Ramachandran plots of the Y107 backbone dihedral angles in the *E. coli* tetramer and dimer simulations, respectively. Red crosses indicate the crystallographic geometries. The orange contour map (or “favoured” region) accounts for 98% of the phi-psi angles analysed by Lovell *et al* [25]. Pale orange contour maps account for 99.95% (“allowed”). Percentages represent the time spent in the 3 regions of the plot.

**Figure 4:**
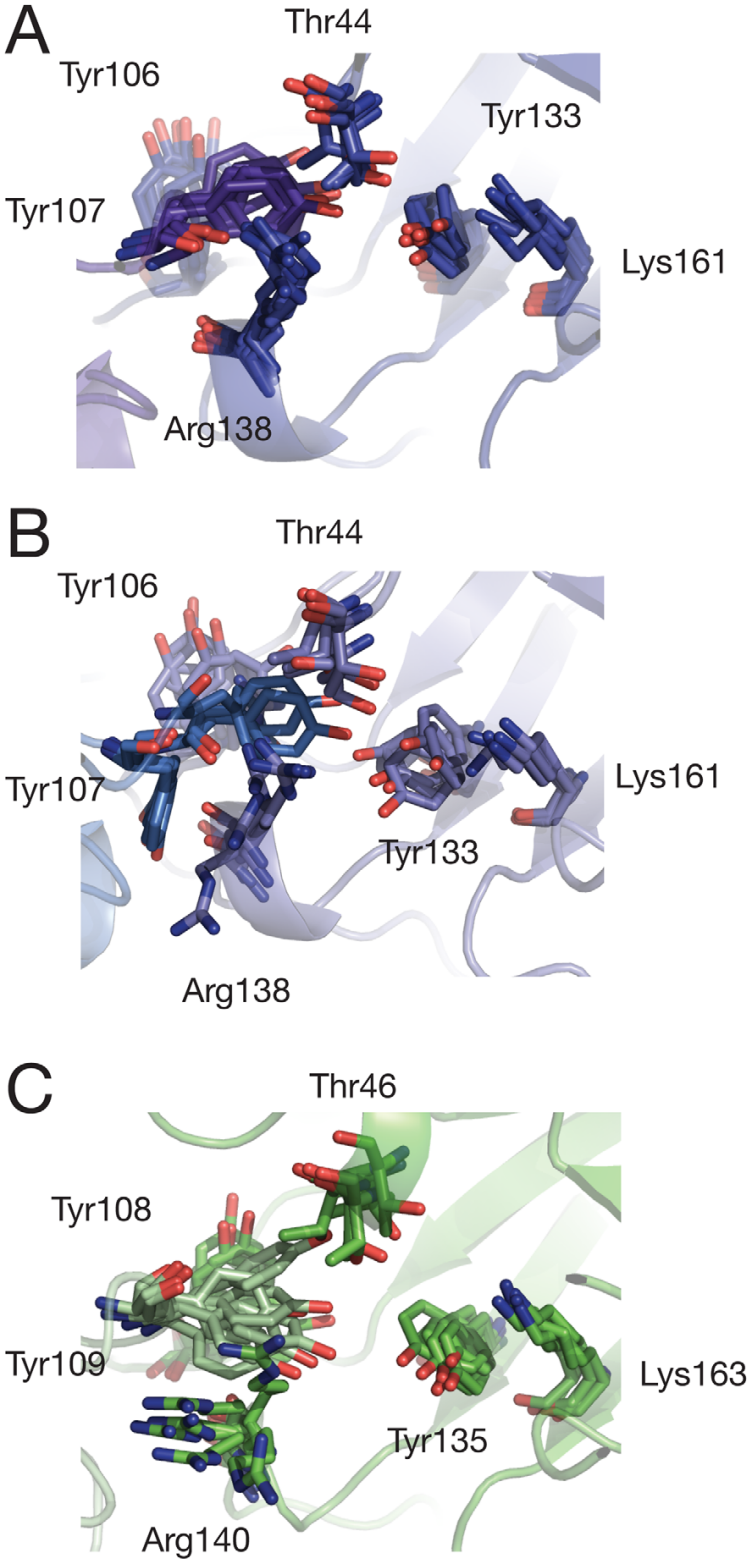
Snapshots of active site residues taken from: (A) *E. coli* tetramer (tet-1), (B) *E. coli* mutant dimer (dim-A), and (C) MR*SA* simulations (mrsa-1). Y107 (*E. coli*)/109 (MR*SA*) is highlighted in purple (A), blue (B) or pale green (C). Snaphots are taken every 100 ns from each trajectory.

To estimate the extent of the active site deformation we calculated the RMSD values (heavy-atoms only) over all the simulations for the eight active residues (T44, Y106, Y133, R138, K161, G186, I203, and Y107 contributed by the adjacent monomer; Figure 1D). Active site residues in the tetramer simulations fluctuate within an RMSD range of 0.8–1.8 Å, with a mean of 1.0 Å, and are relatively stable in their conformation throughout the last 400 ns of the simulations (Figure 3A, grey lines; Figure 4A; Video S3). Conversely, the positions of active site residues in the dimer deviate from the crystal conformation to a much larger degree, with RMSD values varying from an initial 1.0 Å up to 2.8 Å (dim-A) and 3.5 Å (dim-B) towards the end of the simulations (Figure 3A, blue lines; Figure 4B; Video S4). Even though the residues in the dim-A and dim-B active sites show differences in their conformations, they both consistently deviate from the wild-type positions with RMSD values greater than 2 Å over the last 150 ns of the simulations. Our simulations demonstrate that the active sites show more deformation in dimers than in tetramers, where residues show relatively small deviations from their crystal conformation (Figure 4A,B). To estimate potential flexibility in the 8 amino acids composing the active site we calculated the root mean square fluctuations (RMSFs) for the tetramer and dimer simulations (Figure 3B). The results clearly show a general flexibility increase in the dimer active site compared to the tetramer. While the tetramer active site residues display individually low flexibility (RMSF range = 0.4–0.9 Å; Figure 3B and 4A; Video S3), dimer active site residues appear considerably more flexible (RMSF range = 0.6–2.4 Å; Figures 3B and 4B; Video S4). Interestingly, the catalytic residues T44 and Y107 as well as Y106 and R138 contribute most to the increased flexibility within the dimer active site. The remaining residues (Y133, K161, G186, I203) are also more flexible in the dimer compared to the tetramer, although they fluctuate somewhat less (RMSF values<1.0 Å).

The increase in T44 RMSF is due to flipping of its side chain, inverting the positions of the methyl and hydroxyl groups, and results in the transient loss of a hydrogen bond with the hydroxyl group of Y107 (Figure 4A,B). This interaction is known to be essential for activity of the enzyme as it forms part of the catalytic triad [4], [10]. The fluctuations of the hydrophobic patch formed by Y106 and Y107 (both embedded in the tight-dimer interface) contribute the most to the increase in RMSF. The catalytic residue Y107 is of particular interest, since this residue exhibits backbone Φϕ dihedral angles lying in a “disallowed” region of the Ramachandran plot in E. coli DHDPS (wild type and mutants), as well as in other organisms [4], [11]–[13] corresponding to a γ-turn backbone geometry. This suggests that conformational strain is maintained in its backbone, possibly due in part to the backbone carbonyl oxygen bond formed with the guanidino group of R138 [14]. Ramachandran plots for Y107 over the course of the E. coli simulations are shown in Figure 3C (tet-1 and tet-2) and Figure 3D (dim-A and dim-B). Fluctuations in the simulations allow the backbone of Y107 in both tetramers and dimers to explore the Lα geometry; dimers however adopt this geometry for more than half the simulation time. A clear distinctive feature of the dimer simulations is the ability of the Y107 backbone to adopt one “favoured” region (the α region) of the Ramachandran plot that is not populated in the tetramer simulations. This is associated with the loss of the hydrogen bond formed with the R138 guanidino group, resulting in increased movements of the arginine side-chain (Figure 3B, 4B and Video S4). Taken together, these observations provide an explanation for the RMSF increase for this residue, and most likely induce the strain in the backbone of Y107. This is in stark contrast to the tetramer simulations, where the backbone angles of Y107 explore the favoured Lα region of the Ramachandran plot for only 10.9% of the time (Figure 3C).

Recently Pearce *et al.* (2011) [15] have engineered and characterized a monomeric form of DHDPS from the bacterium T. maritama with impaired catalytic function compared to the tetrameric form. The 2.0 Å X-ray structure revealed a well-preserved overall fold and active site geometry compared to its tetrameric form, with the exception of the residues equivalent in E. coli to R138 and Y107 and its surrounding loop [15]. Additionally we find that our dimer simulations reproduce to some extent the backbone conformation of the latter loop of this unique monomeric form, with all Φϕ angles falling in a favoured region of the Ramachandran plot.

The side chains of Y106 and Y107 are also subject to large fluctuations in the dimer simulations. The well-packed hydrophobic stacking formed by the aromatic groups of Y106 and Y107 of both monomers (four tyrosines in total) at the tight-dimer interface in the crystal structures undergoes a dramatic rearrangement resulting in the loss of aromatic stacking in the last 200 ns of simulation. Whereas in the tetramer simulations the Y106 side chain oscillates between conformations that are relatively close to the original crystal structure (Figure 4A and Video S3), the Y107 side chain exhibits largely different conformations towards the end of the dim-A and dim-B simulations (Figure 4B and Supporting Video S4). The latter movements are associated with positional changes of the Y107 hydroxyl group 15 Å away from the two other residues of the catalytic triad (T44, Y133), incompatible with catalysis. We therefore observe in the dimer simulations a critical disruption of the catalytic triad network of hydrogen bonds with the large conformational change of a key residue. As a result, the overall geometry of the catalytic motif is dramatically altered.

In two independent MD simulations, totalling nearly 1 µs, the dynamics of the wild-type E. coli tetramer in the absence of substrate are characterised by ‘near crystal structure’ fluctuations (Figure 3A,B; Figure 4A and Video S3). The overall conformations of the individual monomers, their supra-molecular assembly and the active site only slightly deviate from the structure observed by X-ray crystallography. The dimer simulations show a radically different behaviour: alterations of the monomer arrangement and most importantly critical deformations of the catalytic triad, in particular Y107, potentially rendering the enzyme inactive (Figure 3A,B; Figure 4B and Video S4). If “crystal-like” rigidity is a requirement for a functional enzyme at wild-type levels as shown by the tetramer simulations, the amount of plasticity observed in the isolated dimer, triggered by the change in quaternary structure, provides a straightforward explanation for the dramatic decrease in activity measured [6].

### The naturally occurring and active MRSA DHDPS dimer experiences flexibility, but not active-site deformation

**Figure 5:**
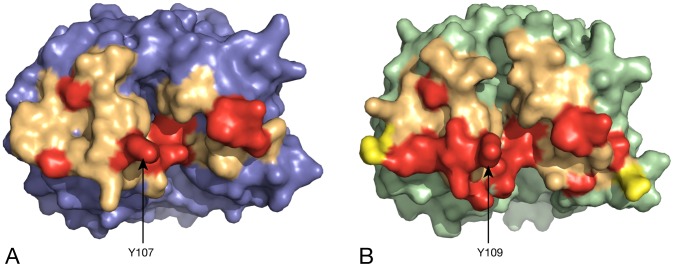
A detailed view of the tight-dimer interface in *E. coli* and MR*SA* DHDPS. Surfaces of both enzymes with the residues involved in the tight-dimer interface represented in light orange. Residues involved in hydrogen bonds are shown in red and in salt-bridges in yellow, as calculated by the PISA server (A) Dimer from *E. coli* wild-type tetramer (PDB ID: 1YXC); (B) MR*SA* wild-type dimer (PDB ID: 3DAQ).

**Figure 6:**
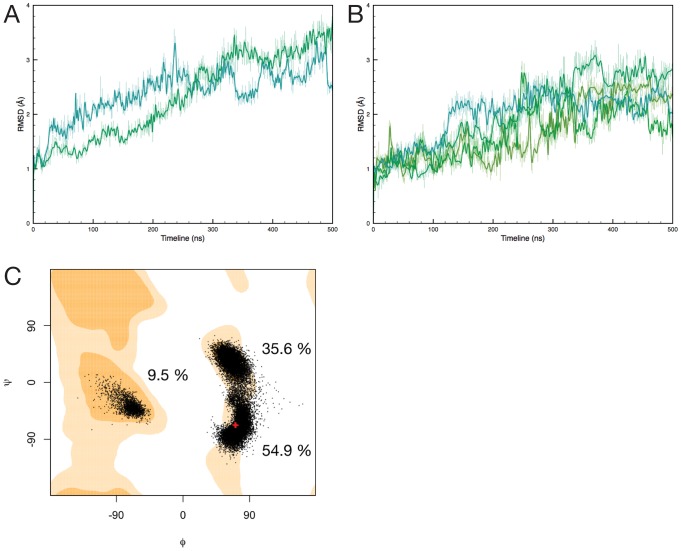
(A) Cα RMSDs over the course of the MR*SA* simulations, for dimers from mrsa-1 (green) and mrsa-2 (turquoise); (B) RMSDs of active site residues over the course of the MR*SA* simulations (4 active sites); (C) Ramachandran plot showing backbone dihedral angles of residue Y109 during the MR*SA* simulations. The red crosses indicate the crystallographic conformations. Percentages represent the time spent in the 3 regions of the plot.

Our simulation data for E. coli DHDPS suggest that conformational fluctuations and flexibility at the active site is a primary cause of the dramatic decrease in enzymatic activity of dimers. The existence of a naturally occurring dimer from the bacterial pathogen MRSA that exhibits comparable activity to the E. coli tetramer is therefore intriguing [8]. Whereas the overall tertiary structures of MRSA and E. coli DHDPS are highly similar (RMSD = 0.9 Å; Figure 1B,C), with only minor reorientations of active site side-chains (Figure 1D), the nature of their tight-dimer interfaces differs (Figure 5). MRSA DHDPS possesses a relatively high number of hydrogen bonds at the tight-dimer interface and two electrostatic interactions that are absent in the E. coli structure, suggesting that it is perhaps less flexible that its E. coli counterpart [8]. We therefore performed two MD simulations of the MRSA DHDPS dimer in the absence of substrate and compared the results to the E. coli DHDPS simulations. The 1.45 Å resolution crystal structure of MR*SA* DHDPS [8] was used as the starting structure for two independent MD simulations of 0.5 µs each in length (denoted mrsa-1 and mrsa-2). Both simulations show a gradual increase in RMSD, which stabilise and reach a plateau at ∼3 Å at ∼300 ns (Figure 6A). The latter corresponds to a rotation of one monomer with respect to the other (Video S6), similar to the E. coli DHDPS dimer (Figure 2B). Active site residues deviate moderately from their crystal conformation over the course of the simulations (Figure 6B and Video S6), reaching a plateau for the last 200 ns, yet somewhat less deviant than the corresponding residues in the E. coli DHDPS dimer (RMSD values of 1.6–3.0 Å compared to 2.2–3.5 Å; Figure 6B).

RMSF values of the active site residues (Figure 4B) are higher than the E. coli tetramer simulations and mostly comparable (within standard deviation) to the E. coli dimer simulations, except for the relatively immobile Y109 (equivalent to Y107 in the E. coli structure). In the mrsa-1/2 simulations the backbone dihedral angles of Y109 populate the same regions as in the dim-A/B simulations (Figure 6C). The simulation time spent in the Φϕ region is similar to dim-A/B, but the proportions are reversed for the γ-turn and Lα regions, consistent with this residue remaining close to the crystal geometry for more than half of the simulation. Furthermore, the extent of the Y109 side chain dynamics is reduced, in contrast to the dim-A/B simulations, and fluctuates near the crystallographic conformation. In addition the aromatic stacking formed with Y108 (equivalent to Y106 in the E. coli structure) as part of the dimer interface remains intact.

**Figure 7:**
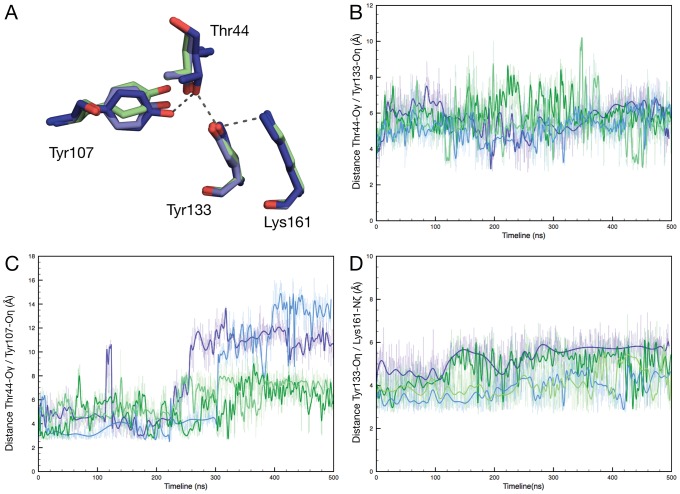
Network of essential active site interactions over the course of the simulations. (A) Electrostatic interactions and residues considered: Wild-type *E. coli* tetramer (dark blue), *E. coli* L197 mutant dimer (light blue), MR*SA* wild-type dimer (green). Only Wild-type *E. Coli* interactions (dashed lines) are shown for clarity. (B) Distance between T44-Oγ and Y133-Oç. (C) Distance between T44-Oγ and Y107-Oç. (D) Distance between K161-Nζ and Y133-Oç. Equivalent MR*SA* residues are T46, Y135, Y109 and K163.

To gain more insight into the potential changes occurring in the active sites we focused on the conserved network of hydrogen bonds present in the catalytic site (Figure 7A). This network is formed by 2 hydrogen bonds between the hydroxyl groups of T44 and Y133 (*E. coli* numbering), and between the hydroxyl groups of T44 and Y107. Point mutation of any of these 3 residues that constitute the catalytic triad results in severely reduced activity [3]. Distances between donor and acceptor atoms were monitored throughout simulations (Figure 7). We find that atoms T44-Oγ/Y133-Oç (Figure 7B) remain in reasonably close contact at a similar average distance of 5.4±1.3 Å and 5.6±1.3 Å in the *E. coli* and the MR*SA* dimers respectively. The hydrogen bond is only transiently formed regardless of the species and broken upon flipping of the T44 side chain. In contrast the distance between T44-Oγ/Y107-Oç shows a marked difference (Figure 7C) following the repositioning of Y107 in the *E. coli* dimer associated with monomer re-arrangement and shown here with a large increase. The relative positions of both side chains seem affected to a smaller extent by rotation in the MR*SA* dimer (average distance is 5.7±1.3 Å) with a small distance increase suggesting weak electrostatic interaction between the hydroxyl groups.

Finally the hydroxyl and ammonium groups of residues Y133 and K161 respectively (Figure 7A, 7D) were monitored. They form an electrostatic interaction in the crystal conformations with a distance of 2.9 (*E. coli* tetramer), 3.4 (MR*SA*) and 3.7 Å (L197Y *E. coli*). Point mutation of substrate binding K161 has been shown to result in largely impaired activity [16]. We find no discernible difference between the dimers with average distances of 4.2±1.0 (*E. coli*) and 4.5±1.1 Å (MR*SA*).

Additionally, in the *E. coli* tetramer simulations all distances were found comparatively shorter and compatible with a tighter and more rigid active site: 4.7±0.9 Å (T44/Y133), 5.1±1.2 Å (T44/Y107) and 3.6±0.6 Å (Y133/K161). We conclude that except for the position of the *E. coli* dimer Y107 the overall active sites architecture and the relative positions of essential side chains remain close (*E. coli* tetramer) or reasonably close (MR*SA*, *E. coli* dimer) to the crystalline state, and are only to a minor extent affected by monomer re-arrangement. Although the functional MRSA DHDPS dimer displays monomer-monomer rotation as well as active site flexibility, unlike the E. coli dimer it does not undergo a similar active site deformation focused around Y109. In contrast, its fluctuations are more distributed amongst the active site residues.

**Figure 8:**
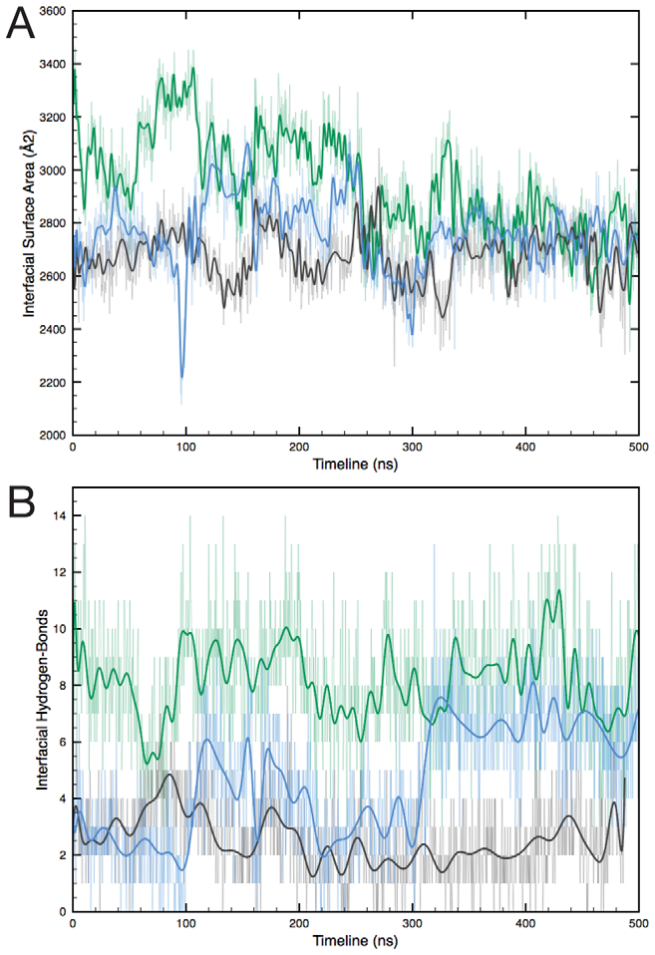
Changes at the tight-dimer interface during simulations. (A). Interfacial surface area buried for both monomers; (B) Number of interfacial hydrogen-bonds (tet-1:black; dim-A: blue; mrsa-1: green). Spline fits (thick lines) of the values (thin lines) are represented for clarity.

Whereas the E. coli DHDPS dimer interface consists of seven hydrogen bonds and three hydrophobic contacts, the larger MRSA DHDPS dimer interface consists of 17 hydrogen bonds and two salt-bridges [8]. We therefore compared and contrasted the nature of the tight-dimer interfaces for *E. coli*. and MR*SA* enzymes. The size of the interfacial area in the E. coli tetramer is stable throughout the simulations. We find that in the MRSA dimer the rotation of the monomers is associated with a reduction in the buried interfacial area, similar in size (∼2700 Å^2^ for two monomers, Figure 8A) to the initial E. coli interface. This does not lead to a decrease in the number of hydrogen bonds (Figure 8B) or salt-bridges, which remains constant. We find however that in the mutant E. coli dimer, while the interfacial buried area is constant, the number of hydrogen bonds contributing to the tight-dimer interface increases with re-orientation of the monomers. In addition we observed the formation of a new salt-bridge per monomer between residues R109 and E246 in dim-A and dim-B, permitted by the new orientation of the monomers. In mrsa-1 and mrsa-2 the equivalent salt-bridge is formed at positions K111 and D247. This suggests that this re-organization of the monomers is more stable than the arrangement found in the crystal state but only compatible with loss of the quaternary structure. Dimer binding energies calculated by the MM-PBSA approach lend support to this hypothesis (Text S1). Disruption of the supra-molecular assembly is associated in E. coli DHDPS with dramatic conformational changes in the active site.

Our simulations show that the MRSA DHDPS enzyme, in the absence of substrate, experiences relatively high flexibility. This is perhaps not unexpected for an enzyme that exists in a monomer-dimer equilibrium in solution [8]. In addition, in contrast to the E. coli dimer, it does not exhibit a localised deformation. We propose that the flexibility observed, without conformational change of critical interface residues such as Y109, preserves the active site geometry and hence enzyme activity.

### Protein dynamics affects specificity towards pyruvate substrate

**Figure 9:**
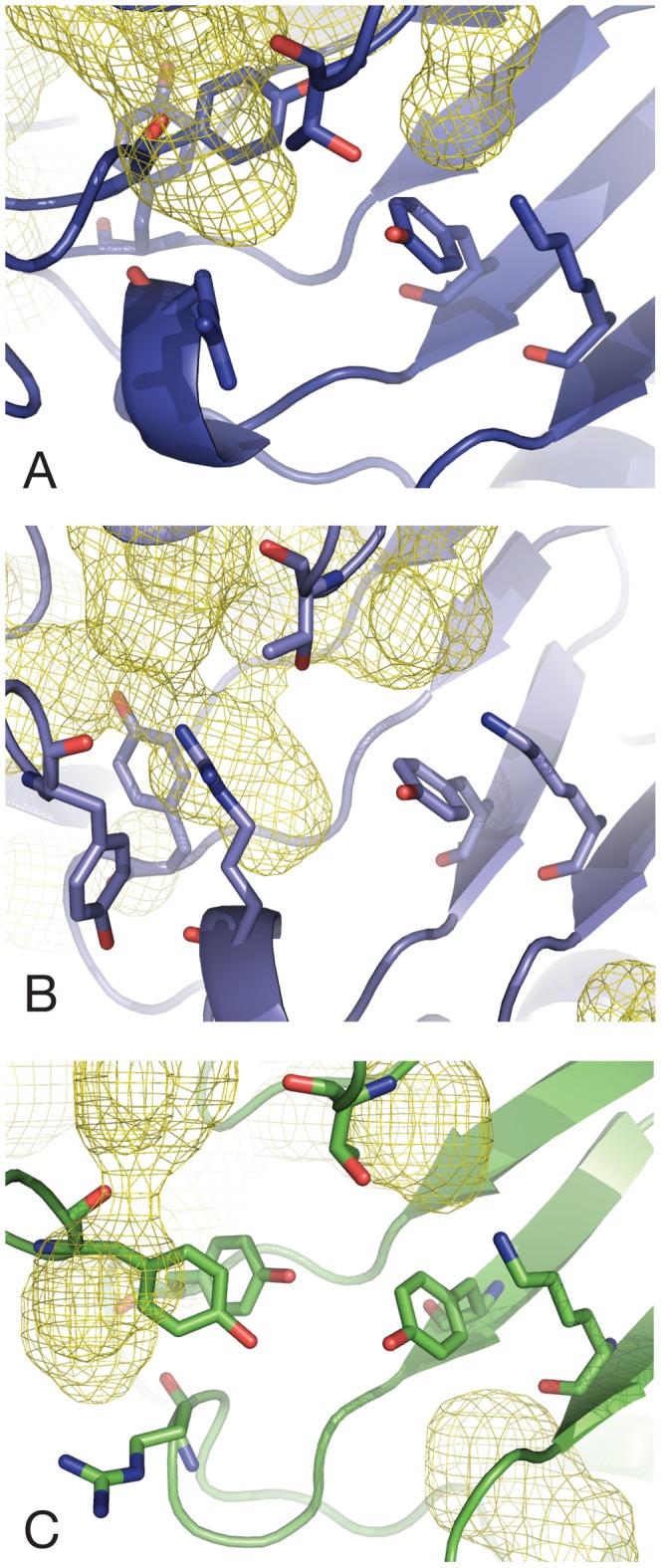
Cavities in DHDPS active sites. (A) Wild-type *E. coli* DHDPS; (B) L197Y *E. coli* engineered dimer; (C) Wild-type MR*SA* DHDPS. Active site cavities are represented as mesh surfaces (yellow) for the last 100 ns of dim-A, tet-1 and mrsa-1.

The mutant dimer L197Y was crystallized in the absence of the substrate pyruvate, with a molecule of α-ketoglutarate trapped in its active site [6]. The latter was not added in the crystallization conditions but rather captured from the expression system. The repositioning of Y107 side chain observed in the L197Y E. coli DHDPS dimer is associated with an enlargement of the active site pocket (Figure 9A and 9B). We propose that the widening of the pocket in the mutant dimer is responsible for allowing the substrate analogue α-ketoglutarate, which is larger than the natural substrate pyruvate, to bind K161 and form a Schiff base before cyclisation, as observed in the crystalline state [6]. This newly formed covalent species acts as a stable inhibitory adducts towards pyruvate, thus explaining the loss of specificity and affinity measured [6]. Following this hypothesis originally formulated by Griffin *et al.* (2008) [6], in MRSA DHDPS the relatively stable positions of all active site residues would prohibit binding and perhaps entry of α-ketoglutarate in the active site. This is reflected by similar affinity for pyruvate and enzymatic activity in both MRSA and wild-type E. coli DHDPS [8].

### Conclusions

Our simulations provide atomistic details of the role of high-level molecular assembly in maintaining optimal activity in the E. coli enzyme. In the mutant *E. coli* dimer we have identified monomer reorientation within the dimer as a major influence on activity, consistent with SAXS data [6]. With the buttressing provided by formation of the dimer of dimers active site geometry is preserved in the tetramer, while in the dimer the enzyme is stripped of a productive catalytic arrangement. Further, simulations of the E. coli mutant dimer reveal a large conformational change of Y107, a key catalytic residue. The wild-type MRSA dimer enzyme is also subject to relatively high flexibility, but in contrast, is counter-balanced by an extended tight-dimer interface, which results in a reasonably well-preserved active site.

Our results suggest that in these two different pathogenic bacterial species, DHDPS optimal activity is achieved by opposing the excess inherent dimer flexibility with two different strategies: in E. coli a higher level quaternary structure buttresses two dimers together while in MRSA an enhanced tight-dimer interface allows preservation of activity. In conclusion, this work supports the hypothesis that a driving force of DHDPS evolution is to optimize intrinsic protein fluctuations to a level compatible with its activity and function [6], [8], [9], [15]. This work also adds to a growing body of evidence linking quaternary structure, protein dynamics and function [17], [18]

## Methods

### Molecular Dynamics simulations

The 1.9 Å resolution X-ray structure of the wild-type E. coli DHDPS tetramer [5] (PDB ID 1YXC) was used for the two independent MD simulations of tetramers (termed tet-1 and tet-2). The dimer simulations employed two different starting structures. In the first case the single mutant enzyme, DHDPS-L197Y, which was solved to 1.7 Å resolution [6] (PDB ID 2OJP), was used (termed dim-A). The coordinates of the bound tetrahedral adduct of its substrate analogue were discarded. Since this may adversely affect the simulation, the second simulation used the dimer structure contained in the asymmetric unit of the native tetramer structure (termed dim-B). Finally, the 1.45 Å resolution crystal structure of DHDPS from MRSA [8] for two independent simulations (mrsa-1, mrsa-2).

In total, we performed 6 independent MD simulations of 3 different DHDPS molecules: two simulations of the native E. coli tetramer (tet-1 and tet-2), two simulations of an E. coli dimer (dim-A and dim-B) and two simulations of the native MRSA dimer (mrsa-1 and mrsa-2). In all simulations, typically 2 to 4 ns were discarded prior to analysis. All simulations employed the same protocol.

#### E. coli DHDPS tetramer simulations

After adding hydrogens, the protein was solvated (TIP3P water model) in a cubic box of initial length 112 Å using VMD [19]. Na^+^/Cl^−^ ions were subsequently added at a concentration of 0.2 M resulting in a chargeless system consisting of 133,245 atoms (38425 water molecules, TIP3P water model). In a first step, the system was minimized (conjugate gradient) for 5000 steps and subjected to 500 ps of simulation with harmonic positional restraints (force constant of 100 kCal(mol Å)^−1^). The system was then submitted to another step of 5000 cycles of minimization followed by 1 ns of simulation with positional restraints of the backbone heavy atoms. Finally, all restraints were relaxed and the system subjected to 5000 steps of minimisation. Random initial velocities were independently assigned to each system (tet-1 and tet-2) and the simulations started.

#### E. coli DHDPS dimer simulations

The first dimer simulation (dim-A) used the high-resolution structure [6] of the engineered dimeric L197Y DHDPS E. coli enzyme (PDB ID 2OJP; 1.7 Å resolution). As this dimer was crystallized with a trapped pyruvate analogue adduct present in the active site, we discarded these coordinates to model the substrate free enzyme (83305 atoms, 24858 water molecules, TIP3P water model, initial cubic box length of 97 Å). As this may create a structural bias in the dim-A simulation, we isolated the symmetric dimer from the tetramer X-ray structure (PDB ID 1YXC, see above) as a different starting structure for the dim-B simulation (83952 atoms, 25012 water molecules, cubic box length of 97 Å). After analysis of the trajectories, both simulations were found to display similar features (see text).

#### MR*SA* DHDPS dimer simulations

Both mrsa-1 and mrsa-2 simulations of the MRSA DHDPS substrate-free enzyme we used the 1.45 Å resolution X-ray structure [8] (PDB ID 3DAQ) as a starting point (84159 atoms, 24973 water molecules, TIP3P water model, initial cubic box length of 97 Å).

All molecular dynamics simulations were performed in NPT conditions. A Langevin thermostat with a damping coefficient of 0.5 ps^−1^ was used to maintain the system temperature (300 K). The pressure was maintained at 1 atm using a Langevin piston barostat. Periodic boundary conditions were applied. The particle mesh Ewald algorithm was used to compute long-range electrostatic interactions. Nonbonded interactions were truncated smoothly between 10 Å and 12 Å. All covalent hydrogen bonds were constrained by the SHAKE algorithm allowing an integration time step of 2 fs. The simulations were run with NAMD 2.7b1 [20] and the CHARMM22 force field with CMAP correction [21], [22].

### Analysis

Structural analysis and measurements were done with the VMD software [19], figures and videos with VMD and PyMol [23]. Cavities were detected with MDpocket [24]; the cavities presented in Figure 9 are the grid points with frequency isovalue 0.3. Ramachandran plots were produced following Lovell et al. [25].

Monomers Cα-RMSDs were calculated with the corresponding minimized crystal structure as a reference. Active sites RMSDs were calculated employing non-hydrogen atoms of the eight residues composing the active site (see text) with the minimized crystal structure as a reference. Active sites residues RMSDs employing the whole monomer as the reference structure displayed an identical trend. Active sites RMSF calculations employed non-hydrogen atoms of the active site as a reference, after removal of the rotation-translation motions by aligning on the first snapshot of the corresponding trajectory. Removal of rotation-translation motions by aligning on the whole monomer yielded an identical trend.

## Supporting Information

Table S1 Binding energies and their components at the beginning of the simulations. Values are averaged over two simulations for each enzyme. Standard deviations are given in brackets.
(DOCX)

Table S2 Binding energies and their components at the end of the simulations. Values are averaged over two simulations for each enzyme. Standard deviations are given in brackets.
(DOCX)

Text S1 Supporting discussion.
(PDF)

Video S1 E. coli wild-type tetramer dynamics.
(MOV)

Video S2 E. coli L197Y mutant dimer dynamics.
(MOV)

Video S3 Active site dynamics of E. coli tetramer.
(MOV)

Video S4 Active site dynamics of E. coli dimer.
(MOV)

Video S5 MRSA wild-type dimer dynamics.
(MOV)

Video S6 Active site dynamics of MRSA dimer.
(MOV)
