# Supplementary material for: Structural and Dynamic Requirements for Optimal Activity of the Essential Bacterial Enzyme Dihydrodipicolinate Synthase
Source: PLoS Comput Biol. 2012 Jun 7;8(6):e1002537. doi: 10.1371/journal.pcbi.1002537 (PMC3369909; doi:10.1371/journal.pcbi.1002537)
Supplement: Table S1 — Binding energies and their components at the beginning of the simulations. Values are averaged over two simulations for each enzyme. Standard deviations are given in brackets. (DOCX) [file pcbi.1002537.s001.docx]

| *Enzyme* | *Energy terms (kcal.mol* ^-1^*)* | | | | | |
| --- | --- | --- | --- | --- | --- | --- |
| *E. coli* dimer | *∆H_MM_* | -203.5 (50.1) | *∆G_solv_* | 97.9 (19.7) | *∆G_bind_* | -125.8 (36.2) |
|  | *∆H_Coulomb_* | -76.2 (45.8) | *∆G_solv-pol_* | 113.6 (20.0) |  |  |
|  | *∆H_vdW_* | -127.3 (12.5) | *∆G_solv-np_* | -15.8 (1.2) |  |  |
|  |  |  |  |  |  |  |
| MR*SA* | *∆H_MM_* | -431.0 (110.5) | *∆G_solv_* | 121.1 (25.5) | *∆G_bind_* | -309.9 (120.7) |
|  | *∆H_Coulomb_* | -302.1 (110.4) | *∆G_solv-pol_* | 138.3 (26.1) |  |  |
|  | *∆H_vdW_* | -128.9 (19.2) | *∆G_solv-np_* | -17.2 (1.4) |  |  |

**Table S1.** Binding energies and their components at the beginning of the simulations. Values are averaged over two simulations for each enzyme. Standard deviations are given in brackets.
