# Supplementary material for: Structural and Dynamic Requirements for Optimal Activity of the Essential Bacterial Enzyme Dihydrodipicolinate Synthase
Source: PLoS Comput Biol. 2012 Jun 7;8(6):e1002537. doi: 10.1371/journal.pcbi.1002537 (PMC3369909; doi:10.1371/journal.pcbi.1002537)
Supplement: Table S2 — Binding energies and their components at the end of the simulations. Values are averaged over two simulations for each enzyme. Standard deviations are given in brackets. (DOCX) [file pcbi.1002537.s002.docx]

| *Enzyme* | *Energy terms (kcal.mol* ^-1^*)* | | | | | |
| --- | --- | --- | --- | --- | --- | --- |
| *E. coli* dimer | *∆H_MM_* | -297.8 (49.5) | *∆G_solv_* | 83.3 (26.1) | *∆G_bind_* | -214.5 (68.3) |
|  | *∆H_Coulomb_* | -191.1 (53.3) | *∆G_solv-pol_* | 98.5 (26.6) |  |  |
|  | *∆H_vdW_* | -106.7 (11.3) | *∆G_solv-np_* | -15.2 (1.1) |  |  |
|  |  |  |  |  |  |  |
| MR*SA* | *∆H_MM_* | -556.3 (97.3) | *∆G_solv_* | 80.5 (23.5) | *∆G_bind_* | -475.9 (106.5) |
|  | *∆H_Coulomb_* | -455.8 (97.7) | *∆G_solv-pol_* | 96.1 (23.9) |  |  |
|  | *∆H_vdW_* | -100.6 (12.5) | *∆G_solv-np_* | -15.6 (1.3) |  |  |

**Table S2.** Binding energies and their components at the end of the simulations. Values are averaged over two simulations for each enzyme. Standard deviations are given in brackets.
