# Supplementary material for: Structural and Dynamic Requirements for Optimal Activity of the Essential Bacterial Enzyme Dihydrodipicolinate Synthase
Source: PLoS Comput Biol. 2012 Jun 7;8(6):e1002537. doi: 10.1371/journal.pcbi.1002537 (PMC3369909; doi:10.1371/journal.pcbi.1002537)
Supplement: Text S1 — Supporting discussion. (PDF) [file pcbi.1002537.s003.pdf]

Supporting Material

Supporting Discussion: Dimer binding energy

To estimate the change in binding energies before and after monomer rearrangement monomer-monomer binding energies ΔG_{bind} were estimated with the MM-PBSA approach for both *E. coli* and MR*SA* dimers. ΔG_{bind} was calculated from the difference between the complexed and the separate (each monomer taken individually) energies. The energies and their components were computed for both species for the first 150 ns of simulation, where the molecule is characterized by 'near crystal' fluctuations (Table S1), and for the last 150 ns of simulation (after monomer/monomer rotation, Table S2).

The free energy G_{tot} of each molecule was calculated following:

$$G_{tot} = H_{MM} + G_{solv} - TS_{conf},$$

and ΔG_{bind} is given by:

$$\Delta G_{bind} = G_{tot}^{dimer} - G_{tot}^{monomer1} - G_{tot}^{monomer2}$$

H_{MM} is the sum of the force field energy terms. Since we calculate the binding energies from the complexed trajectories the energy differences for the bond, angle and dihedrals terms are equal to zero. H_{MM} is thus the sum of the Coulomb electrostatic $H_{Coulomb}$ and van der Waals (vdW) H_{vdW} energies. G_{solv} is the solvation free energy. The latter is here split into two components: the non-polar contribution ($G_{solv\text{-}np}$) and the polar contribution ($G_{solv\text{-}pol}$, estimated by the PBSA method).

Because of the low prediction accuracy and large computational cost, the conformational entropy term was omitted. However, for two dimers that experience similar monomer rearrangement, one could perhaps expect that the conformational entropy difference upon binding be reasonably similar.

Additional computational details are given at the end of the section.

For the first 150 ns of simulation (Supplementary table 1), one can note a marked difference of the ΔH_{MM} term (~ 230 kcal.mol^{-1}) between the enzymes. This can be solely attributed to the Coulomb electrostatic interaction energies ($\Delta H_{Coulomb}$) considerably more favourable in the MR*SA* than in the *E. coli* enzyme. This is in line with the observation that the MR*SA* interface is enriched in hydrogen-bonds and one salt bridge (with more interfacial charged interactions) compared to the *E. coli* dimer. The polar solvation energy component $\Delta G_{solv\text{-}pol}$ follows the inverse relationship, being more favourable in the *E. coli* enzyme. This is not unexpected that a more charged interface (MR*SA* DHDPS) would have a less favourable polar desolvation energy upon binding than a less polar or charged interface (*E. coli* DHDPS). We find the van der Waals and non polar solvation energy components identical for both enzymes, which most likely mirrors their similarity in interfacial size and shape. Overall, MR*SA* DHDPS displays features of a stronger interface with a more favourable ΔG_{bind} largely driven by electrostatic interactions.

After rotation of the monomers (Table S2), ΔH_{vdW} is less favourable and comparable in both enzymes (as expected from similar interfacial surface area, Figure 8A), in favour of Coulomb energies. They are found stronger than at the beginning of the simulations with a gain of ~ 120 (*E. coli*) and ~ 150 kcal.mol^{-1} (MR*SA*). The solvation free energy is similar for both enzymes and leads to total binding energies much larger than for the first 150 ns of simulation. This is consistent with the hypothesis that the enzymes interface becomes stronger after monomer rearrangement.

We note that the values computed are characterized by large standard deviations particularly for $\Delta H_{Coulomb}$, $\Delta G_{solv\text{-}pol}$ and consequently for ΔG_{bind}. Along with an incomplete thermodynamic cycle this restrains us from making definite conclusions. However, the amplitude of the total binding energies lend further support to our hypothesis that monomer re-organization is associated with stronger monomer interactions, largely accounted for by the Coulomb term as reflected by an increase of hydrogen bonds at *E. coli* mutant dimer interface and one salt bridge in both enzymes (see text and Figure 8B).

Computational details

Energies were computed for all simulations every 500ps employing PDB2PQR 1.8[1] & APBS 1.3[2] for the Poisson-Boltzmann contribution. The force field components employed NAMD 2.8[3] with the CHARMM22/CMAP correction force field[4; 5]. For APBS calculations, CHARMM radii and charges were used, the grid spacing was 0.5 Å, the solvent dielectric 80.0, the protein dielectric 1.0, the cubic spline window was set to 0.3 and the solvent radius to 1.4 Å. No counter-ions were used. $G_{solv\text{-}np}$ was calculated from:

$G_{solv\text{-}np} = \gamma SASA + \beta$, with the surface tension γ and the offset β set to the values of 0.00542 kcal.mol^{-1}Å^{-2} and 0.92 kcal.mol^{-1}, in accord with the MM-PBSA approach[6].

Supporting References

1. Dolinsky, T. J., Czodrowski, P., Li, H., Nielsen, J. E., Jensen, J. H., Klebe, G. & Baker, N. A. (2007). PDB2PQR: expanding and upgrading automated preparation of biomolecular structures for molecular simulations. *Nucleic Acids Res* **35**, W522-5.
2. Baker, N. A., Sept, D., Joseph, S., Holst, M. J. & McCammon, J. A. (2001). Electrostatics of nanosystems: application to microtubules and the ribosome. *Proc Natl Acad Sci U S A* **98**, 10037-41.
3. Phillips, J. C., Braun, R., Wang, W., Gumbart, J., Tajkhorshid, E., Villa, E., Chipot, C., Skeel, R. D., Kale, L. & Schulten, K. (2005). Scalable molecular dynamics with NAMD. *J Comput Chem* **26**, 1781-802.
4. MacKerell, A. D., Bashford, D., Bellott, M., Dunbrack, R. L., Evanseck, J. D., Field, M. J., Fischer, S., Gao, J., Guo, H., Ha, S., Joseph-McCarthy, D., Kuchnir, L., Kuczera, K., Lau, F. T. K., Mattos, C., Michnick, S., Ngo, T., Nguyen, D. T., Prodhom, B., Reiher, W. E., Roux, B., Schlenkrich, M., Smith, J. C., Stote, R., Straub, J., Watanabe, M., Wiorkiewicz-Kuczera, J., Yin, D. & Karplus, M. (1998). All-atom empirical potential for molecular modeling and dynamics studies of proteins. *Journal of Physical Chemistry B* **102**, 3586-3616.
5. Mackerell, A. D., Jr., Feig, M. & Brooks, C. L., 3rd. (2004). Extending the treatment of backbone energetics in protein force fields: limitations of gas-phase quantum mechanics in reproducing protein conformational distributions in molecular dynamics simulations. *Journal of computational chemistry* **25**, 1400-15.
6. Chong, L. T., Duan, Y., Wang, L., Massova, I. & Kollman, P. A. (1999). Molecular dynamics and free-energy calculations applied to affinity maturation in antibody 48G7. *Proc Natl Acad Sci U S A* **96**, 14330-5.
7. Griffin, M. D., Dobson, R. C., Pearce, F. G., Antonio, L., Whitten, A. E., Liew, C. K., Mackay, J. P., Trewhella, J., Jameson, G. B., Perugini, M. A. & Gerrard, J. A. (2008). Evolution of quaternary structure in a homotetrameric enzyme. *J Mol Biol* **380**, 691-703.
